# Metabolite profiling of saliva for the discrimination of Behcet’s disease, Sjögren’s syndrome, and recurrent aphthous stomatitis

**DOI:** 10.1371/journal.pone.0351403

**Published:** 2026-06-09

**Authors:** Sooah Kim, Ming Zhang, Joong Kyong Ahn, Jiwon Hwang

**Affiliations:** 1 Department of Environment Science & Biotechnology, Jeonju University, Jeonju, Republic of Korea; 2 Guangxi College and University Key Laboratory of High-Value Utilization of Seafood and Prepared Food in Beibu Gulf, Qinzhou Key Laboratory of Food Flavor Analysis and Control, Beibu Gulf University, Qinzhou, China; 3 Division of Rheumatology, Department of Internal Medicine‌‌, Kangbuk Samsung Hospital, Sungkyunkwan University School of Medicine, Seoul, Republic of Korea; 4 Division of Rheumatology, Department of Internal Medicine, Kyung Hee University Hospital, Kyung Hee University College of Medicine, Seoul, Republic of Korea; University of the Republic Uruguay: Universidad de la Republica Uruguay, URUGUAY

## Abstract

Oral manifestations can be the initial sign of systemic diseases such as Behcet’s disease (BD) and Sjogren’s syndrome (PSS). Their frequency and morphology vary widely, and recurrent aphthous stomatitis (RAS) often mimics BD ulcers, complicating differentiation on clinical grounds. Nonspecific aphthoid lesions are also seen in PSS. This study aimed to identify distinctive metabolic patterns in saliva that could discriminate BD, PSS, and RAS using global metabolite profiling. Saliva samples were collected from 43 patients (BD, n = 24; PSS, n = 10; RAS, n = 9) after fasting and abstaining from oral activities for at least 90 minutes. Gas chromatography-mass spectrometry (GC/MS) was employed for metabolite profiling. Principal component analysis (PCA) and hierarchical clustering analysis (HCA) were used to distinguish groups. Variable importance in projection (VIP) scores were calculated from partial least squares discriminant analysis (PLS-DA), and ANOVA was applied to compare metabolite abundance. Forty-two metabolites were identified and categorized into amino acids, organic acids, sugars, sugar alcohols, and others. PCA and HCA demonstrated clear dis-crimination among BD, PSS, and RAS. Metabolites with VIP > 1 included malonic acid sorbitol, pyroglutamic acid, aspartic acid, decanoic acid, hexadecenoic acid, and L-proline. Notably, malonic acid and aspartic acid were significantly elevated in BD compared to PSS and RAS, suggesting their diagnostic potential. Global salivary metabolite profiling by GC/MS provides distinct metabolic signatures enabling discrimination among BD, PSS, and RAS. This approach may enhance understanding of oral mucosal manifestations in systemic autoimmune diseases.

## Introduction

Recurrent Aphthous Stomatitis (RAS) is a common ulcerative condition of the oral mucosa, and its minor form typically resolves spontaneously within 7–14 days [1]. While most patients are otherwise healthy, severe forms such as major aphthous ulcers may present with multiple or extensive lesions confined to the oral cavity [2]. Importantly, RAS-like ulcerations can also occur as mucocutaneous manifestations of systemic autoimmune or inflammatory disorders such as Behçet’s disease (BD) and Sjögren’s syndrome (PSS) [3–5]. Behçet’s disease is a chronic, relapsing, multisystem inflammatory disorder characterized by recurrent mucocutaneous lesions and variable organ involvement [6]. Oral ulcers are among the hallmark features of BD and remain central to clinical diagnosis [7,8]. Previous studies have demonstrated that major aphthous ulcers are more frequent and oral involvement is more extensive in BD than in idiopathic RAS [9,10]. Diagnostic uncertainty frequently arises when recurrent oral ulceration is the initial or sole manifestation of BD, especially in the absence of other characteristic systemic features, underscoring the need for reliable markers for differential diagnosis [11]. Sjögren’s syndrome is a systemic autoimmune disease that predominantly affects middle-aged women and is characterized by lymphocytic infiltration of exocrine glands [12]. Because oral lesions, including recurrent oral ulcerations, have also been reported in PSS, differentiating RAS, BD, and PSS may be particularly challenging when systemic features are not yet fully developed [13,14]. Previous studies have compared the oral manifestations of BD and idiopathic RAS, while others have described oral lesions in PSS or salivary metabolic alterations in RAS or PSS. However, direct comparative data across RAS, BD, and PSS remain limited [15–17].

Metabolomics, the comprehensive analysis of small-molecule metabolites within biological systems, has emerged as a powerful tool for elucidating disease mechanisms and identifying diagnostic biomarkers. Metabolites measurable in physiological fluids such as blood, urine, and saliva can provide real-time insights into dynamic metabolic alterations [18]. Saliva, in particular, offers a non-invasive, easily accessible biofluid with growing diagnostic utility for biomarker discovery [19–21]. Despite growing interest in salivary metabolomics, its potential value for distinguishing RAS, BD, and PSS remains unclear. Therefore, this study aimed to characterize salivary metabolite signatures across these three conditions to assess their potential value for differential diagnosis and to identify candidate biomarkers relevant to BD and PSS.

## Materials and methods

### *Study subjects and sample collection*‌‌

The study subjects include patients with RAS, BD, and PSS. All diagnoses were established by board-certified rheumatologists. BD was defined according to the Japanese diagnostic criteria for Behcet’s disease [22], and PSS was classified according to the 2016 American College of Rheumatology (ACR)/European League Against Rheumatism (EULAR) classification criteria for primary Sjögren’s syndrome [23]. RAS was defined as recurrent oral ulceration without systemic features suggestive of BD or PSS and without fulfillment of corresponding diagnostic or classification criteria. Clinical information of patients, including age, gender, medical history, including the onset of oral symptoms, and laboratory data, was collected after written informed consent was obtained. Antinuclear antibody (ANA) and anti-Ro antibody results were obtained from routine clinical laboratory testing in electronic medical records. Both tests were performed on serum samples, and positivity was determined according to the reference ranges and cut-off values used by the institutional laboratory at the time of testing. The study was performed in accordance with the 1964 Helsinki Declaration and was approved by the Institutional Review Board of Samsung Changwon Hospital (SCMC 2020-03-003). All participants were adults and no minors were included in this study. The written informed consent was obtained from each participant prior to saliva collection. Participant recruitment and saliva collection were conducted from the date of IRB approval (April, 2020) until February, 2023.

Saliva samples were collected from each participant in the morning. Prior to sample collection, participants will be instructed to abstain from eating, drinking, or engaging in oral hygiene activities for at least 90 minutes. Standard saliva collection devices will be used to collect oral saliva, and samples will be stored at −80 °C until analysis, to ensure sample preservation.

This study was designed as an exploratory, hypothesis-generating salivary metabolomics study rather than a confirmatory study. BD and PSS are relatively uncommon systemic immune-mediated diseases, whereas RAS is more common but clinically heterogeneous and was included as a disease comparator because of its overlapping oral manifestations. Given this exploratory design, a formal sample size calculation based on a prespecified effect size and statistical power was not performed. Instead, the sample size was determined pragmatically by considering the epidemiological characteristics of the study diseases, the feasibility of recruiting well-characterized participants, the availability of saliva samples suitable for GC/MS-based metabolomic profiling, and sample sizes used in previous comparable metabolomics studies [24–26].

### Sample preparation and metabolite analysis

Sample preparation was performed by modifying a previously established protocol [27]. Briefly, saliva samples (500 μL) were subjected to rotary vacuum drying, followed by extraction using a solvent mixture (methanol: chloroform: distilled water in a ratio of 14: 4: 2.85). The resulting supernatant was transferred to a new centrifuge tube and subjected to rotary vacuum drying. The dried extracts were promptly derivatized by mixing 10 µL of methoxyamine hydrochloride in pyridine (40 mg/mL) for 90 min at 30°C, followed by 45 µL of N-methyl-N-(trimethylsilyl) trifluoroacetamide (MSTFA) for 30 min at 30°C. To ensure data quality, a mixture of fatty acid methyl esters (C8–C30) was utilized to monitor retention time shifts and ensure stable performance of the GC/MS system. All samples under-went analysis within 24 hours of derivatization. To check the reliability of our measurements, a set of 32 known compounds including alanine, ribitol, putrescine, and cholesterol was analyzed before and after the sample runs [28].

Metabolite analysis was conducted using Gas Chromatography-Mass Spectrometry (GC/MS) (Shimadzu QP2010 Plus, Shimadzu) equipped with an Rtx-5sil column (30 m × 0.25 mm i.d. × 0.25 µm film thickness). The oven temperature was initially set at 50°C for 2 min, then programmed to 200°C at a rate of 5 °C/min, held for 5 min, further programmed to 330 °C at a rate of 10 °C /min, and finally held for 5 min. The interface and ion source temperatures were maintained at 280°C and 250°C, respectively. Mass spectra were acquired in the mass range of 85–600 m/z at an ionization voltage of 70 eV.

### Statistical analysis

As the statistical analyses of metabolite profiles of saliva from study groups (BD, PSS, and RAS), univariate analysis, principal component analysis (PCA), and hierarchical clustering analysis (HCA) were performed to assess group separation. Across three groups, variable importance for projection (VIP) scores were computed from partial least squares discriminant analysis (PLS-DA), and ANOVA tests were employed to compare the abundance of metabolite. Data processing involved deconvolution using the Auto-mated Mass Spectral Deconvolution and Identification System (AMDIS; version 3.2). Metabolite identification was achieved using the Golm Metabolome Database (GMD). Sub-sequent data refinement utilized SpectConnect with an elution threshold of 1 min and support threshold of 70%. The processed data underwent normalization using sum normalization and range scaling.

## Results

### Patient characteristics

Baseline characteristics of the study participants are summarized in Table 1. The mean age was comparable across groups. Although female predominance was evident in the BD (75.0%) and PSS (90.0%) groups, the RAS group included a lower proportion of female patients (44.4%), with a borderline difference among groups (p = 0.079). ANA positivity differed significantly among groups (p < 0.001). Although ANA is not included in the 2016 ACR/EULAR classification criteria for PSS, it is a basic screening test for autoimmune disease. In contrast, all PSS patients were positive for anti-Ro antibodies, consistent with the serologic item directly relevant to the classification criteria. HLA-B51 testing was not performed in all participants. Among those tested, HLA-B51 positivity showed a statistically significant intergroup difference (p = 0.040).

**Table 1:** Baseline characteristics according to patient groups.

	BD	PSS	RAS	p-value
Number	24	10	9	
Age, years	49.1 ± 9.7	57.0 ± 10.8	56.4 ± 16.8	0.119
Female (%)	18 (75.0)	9 (90.0)	4 (44.4)	0.079
Oral symptoms, including ulcer or dryness, or both (%)	23 (95.8)	8 (80.0)	9 (100)	0.176
Oral symptom onset to diagnosis, months, median [range]	12.0 [0–390]	70.0 [4–317]	52 [1–335]	0.780
ANA positivity/tested (%)	0/24 (0)	6/10 (60)	1/9 (11.1%)	<0.001
HLA-B51 positivity/tested (%)	13/17 (76.5)	0/1 (0)	3/9 (33.3%)	0.040

### Identification of metabolite from saliva samples

A total of 43 salivary samples of study groups analyzed using GC/MS. After the raw data were processed with BinBase, 42 metabolites were identified. These 42 metabolites were classified based on their chemical structures into different classes, including amino acids (9), organic acids (16), sugars and sugar alcohols (6), and others (11) (Table 2). The identified metabolites in this study were involved in various metabolic pathways within tissues, such as biosynthesis of unsaturated fatty acids, glutathione metabolism, amino-acyl-tRNA biosynthesis, and arginine and proline metabolism.

**Table 2:** Identified salivary metabolites detected by GC–MS.

Chemical class	Metabolite	quant mass (m/z)	KEGG id	PubChem id
Amino acid	Aspartic acid	232	C00049	5960
	Cysteine	220	C00097	594
	Glycine	102	C00037	750
	L-Leucine	158	C00123	6106
	L-Proline	142	C00148	145742
	L-Serine	73	C00065	5951
	L-valine	144	C00183	6287
	Ornithine	142	C00077	6262
	Pyroglutamic acid	156	C01879	7405
Organic acids	4-Hydroxybenzoic acid	267	C00156	135
	Decanoic acid	117	C01571	2969
	Dodecanoic acid	117	C02679	3893
	Eicosanoic acid	73	C06425	10467
	Glutaric acid	147	C00489	743
	Hexadecanoic acid	117	C00249	985
	Lactic acid	73	C00256	61503
	Linoleic acid	337	C01595	5280450
	Malic acid	233	C00149	222656
	Malonic acid	147	C00383	867
	Nonanoic acid	117	C01601	8158
	Octadecanoic acid	73	C01530	5281
	Oleic acid	339	C00712	445639
	Suberic acid	73	C08278	10457
	Succinic acid	247	C00042	1110
	Tetradecanoic acid	117	C06424	11005
Sugar	Fructose	307	C00095	107428
	Glucopyranose	204	C00221	64689
	Glucose	160	C00031	5793
	Maltose	361	C00208	439186
	Melezitose	361	C08243	92817
	Sorbitol	103	C00794	5780
Others	3-Hydroxypropanoic acid	73	C01013	68152
	Bornesitol	305	C03659	440078
	Cholesterol	129	C00187	5997
	Glycerol 3-phosphate	299	C00093	754
	myo-Inositol	305	C00137	892
	Octadecanol	327	D01924	8221
	Phosphoric acid	314	C00009	1004
	Putrescine	174	C00134	1045
	Triethanolamine	118	C06771	7618
	Urea	147	C00086	1176
	Uridine	113	C00299	6029
				

### Discrimination of metabolites

**Fig 1:**
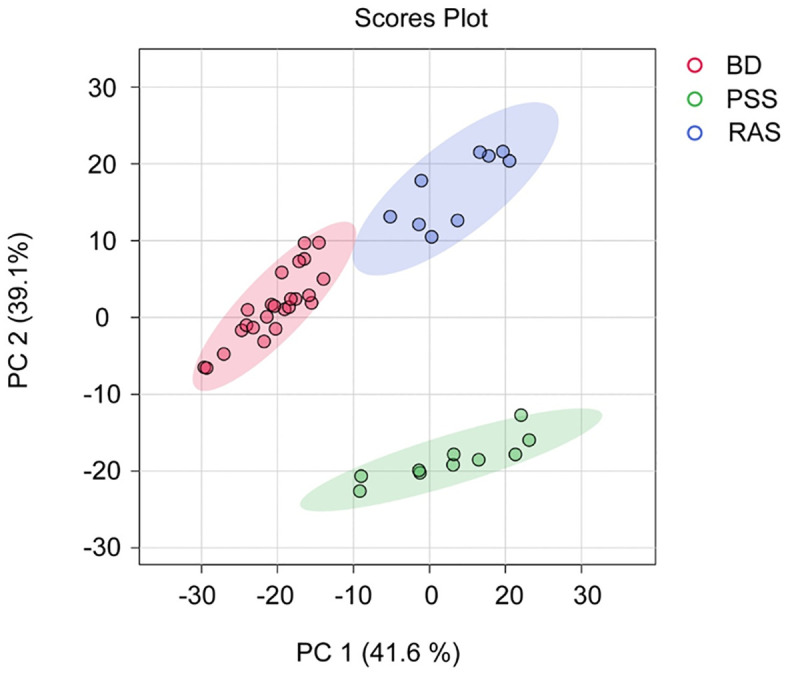
Principal component analysis (PCA) score plot of salivary metabolites for Behçet’s disease (BD), Sjögren’s syndrome (PSS), and recurrent aphthous stomatitis (RAS).

As shown in Fig 1, a clear separation between 3 different diseases was observed, with the first principal component (PC1) accounting for 41.6% of the variance and the second principal component (PC2) explaining 39.1%. The selected 20 metabolites with their highest loading scores, which represent how each metabolite contributed to the new variables generated using the PCA model, are listed in Table 3.

**Table 3:** Twenty metabolites with their high absolute loading scores on PC1 and PC2 obtained by PCA.

PC1		PC2	
Metabolite	Loading score	Metabolite	Loading score
Malonic acid	−0.638	Cholesterol	0.521
Pyroglutamic acid	0.307	Malonic acid	−0.416
Sorbitol	0.305	Aspartic acid	0.345
Aspartic acid	−0.298	L-Proline	−0.323
L-Proline	0.257	Glycine	−0.254
Glycine	0.222	3-Hydroxypropanoic acid	0.243
Putrescine	0.151	Sorbitol	0.202
Urea	0.126	Hexadecanoic acid	0.193
Linoleic acid	0.122	Putrescine	−0.178
Oleic acid	0.112	Decanoic acid	−0.119
Melezitose	0.103	Urea	−0.118
Octadecanoic acid	0.095	Octadecanoic acid	0.094
Hexadecanoic acid	0.093	Nonanoic acid	−0.088
Tetradecanoic acid	0.092	Octadecanol	−0.079
Lactic acid	0.092	Cysteine	0.074
L-Leucine	0.087	myo-Inositol	0.072
Suberic acid	0.076	Glucose	−0.071
Phosphoric acid	0.075	L-Serine	−0.067
Cholesterol	−0.075	Succinic acid	−0.064
Succinic acid	0.074	Lactic acid	0.062

Of the identified 42 metabolites, 17 including pyroglutamic acid, sorbitol, L-proline, glycine, putrescine, urea, linoleic acid, oleic acid, and melezitose, positively contributed to PC1. In contrast, 7 metabolites, including malonic acid, aspartic acid, cholesterol, 3-hydroxypropanoic acid, myo-inositol, decanoic acid, and dodecanoic acid, negative contributions to PC1. Then, HCA was performed on 42 metabolites extracted from saliva samples of various diseases. Clustering similarity was assessed using the Pearson correlation coefficient and the average linkage method. Each vertical column in the analysis represents an individual sample, while each horizontal axis represents the metabolites under consideration.

**Fig 2:**
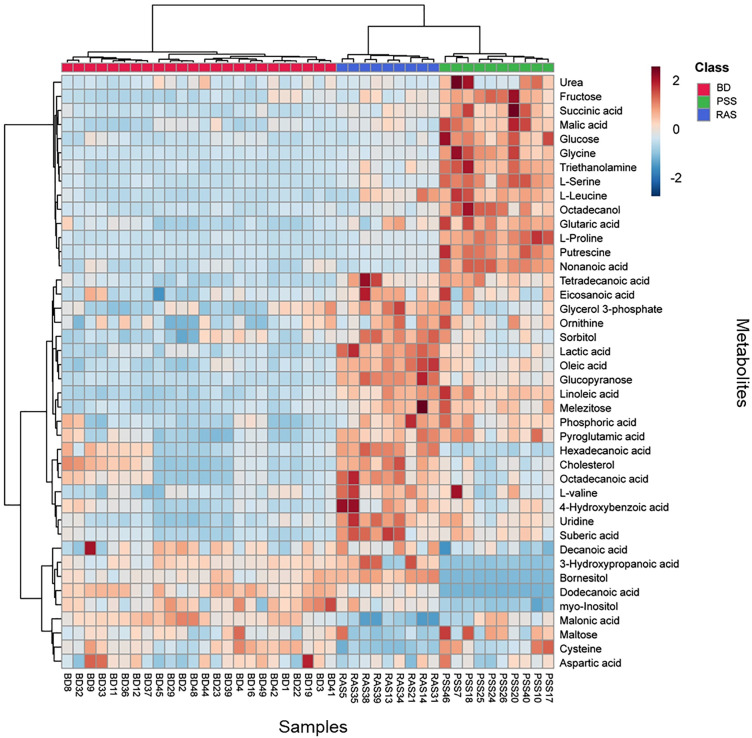
Hierarchical clustering analysis (HCA) of salivary metabolites from Behçet’s disease (BD), Sjögren’s syndrome (PSS), and recurrent aphthous stomatitis (RAS).

**Fig 3:**
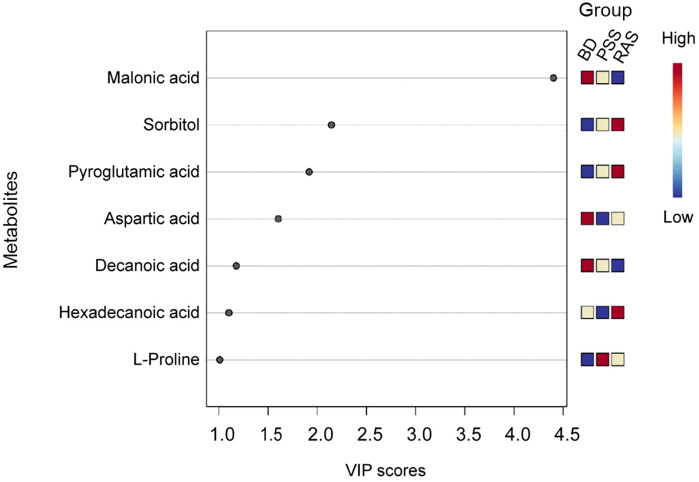
Variable importance for projection (VIP) scores plot derived from partial least squares-discriminant analysis (PLS-DA). The heat-scale bar indicates relative metabolite abundance (red = high, blue = low) across BD, PSS, and RAS.

As shown in Fig 2, it can be observed that the content of urea, fructose, succinic acid, malic acid, glucose, glycine, triethanolamine, L-serine, L-leucine, octadecanol, glutaric acid, L-proline, putrescine, nonanoic acid, dodecanoic acid, 3-hydroxypropanoic acid, cholesterol, aspartic acid, and myo-inositol is higher in PSS compared to RAS and BD. These findings indicate significant inter-disease differences in salivary metabolite levels. Notably, among these metabolites, aspartic acid and L-proline were also identified as key discriminatory metabolites in the PLS-DA analysis (Fig 3), further supporting their potential as biomarkers for discriminating between RAS, BD, and PSS.

### Identification of salivary biomarker for recurrent oral symptoms

In the comparison of metabolite abundance differences among RAS, BD, and PSS, ANOVA and post-hoc tests were employed, and differentially identified metabolites were selected based on p < 0.05. The results revealed that 41 of 42 compounds exhibited significant differences among the three diseases (p < 0.05) (Table 4). Malonic acid and aspartic acid showed significantly higher levels in BD compared to PSS and PAS, while putrescine, nonanoic acid, glycine, glucose, octadecanol, malic acid, and urea exhibited significantly higher levels in PSS compared to BD and RAS. Octadecanoic acid, 4-hydroxybenzoic acid, glycerol 3-phosphate, and cysteine demonstrated significantly higher levels in RAS compared to BD and PSS. These metabolites may serve as potential biomarkers for distinguishing among the three diseases. PLS-DA was utilized to compute VIP scores across three groups. Differentially identified metabolites were chosen based on VIP scores greater than 1, leading to the identification of seven metabolites: Malonic acid, sorbitol, pyroglutamic acid, aspartic acid, decanoic acid, hexadecanoic acid, and L-Proline (Fig 3). Among these metabolites, malonic acid, aspartic acid, and decanoic acid were higher in BD, L-proline was increased in PSS, while sorbitol, pyroglutamic acid, and hexadecanoic acid showed the highest levles in RAS, indicating distinct metabolic alterations across the three diseases. Collectively, these results indicate that specific salivary metabolites exhibit disease-dependent changes, with malonic acid and aspartic acid elevated in BD, L-proline increased in PSS, and cysteine elevated in RAS, suggesting their potential as non-invasive biomarkers for discriminating among RAS, BD, and PSS. Several metabolites such as malonic acid, aspartic acid, putrescine, and L-proline were found to be associated with the regulation of inflammation, immune response, and tissue repair [29–35]. In particular, malonic acid and aspartic acid in BD are linked to oxidative stress and immune regulation [26–28], and putrescine and L-proline in PSS reflect anti-inflammatory responses and tissue repair [29–32]. Therefore, salivary metabolomics could serve as a valuable diagnostic tool for identifying recurrent oral disorders and for monitoring disease progression in clinical practice. It may be particularly helpful in the early differentiation of patients presenting with nonspecific oral ulcers, where clinical symptoms overlap among BD, PSS, and RAS, potentially supporting timely diagnosis and reducing the need for invasive procedures such as biopsy.

**Table 4:** ANOVA and post hoc tests for salivary metabolites among BD, PSS, and RA.

Metabolite	p value	Post hoc tests
Malonic acid	< 0.05	BD – PSS; BD – RAS; PSS – RAS
Aspartic acid	< 0.05	BD – PSS; BD – RAS; RAS – PSS
Cholesterol	< 0.05	BD – PSS; RAS – BD; RAS – PSS
Hexadecanoic acid	< 0.05	BD – PSS; RAS – BD; RAS – PSS
3-Hydroxypropanoic acid	< 0.05	BD – PSS; RAS – BD; RAS – PSS
myo-Inositol	< 0.05	BD – PSS; RAS – PSS
Dodecanoic acid	< 0.05	BD – PSS; RAS – PSS
Decanoic acid	< 0.05	BD – RAS; PSS – RAS
Putrescine	< 0.05	PSS – BD; PSS – RAS
Nonanoic acid	< 0.05	PSS – BD; PSS – RAS
Glycine	< 0.05	PSS – BD; PSS – RAS
Glucose	< 0.05	PSS – BD; PSS – RAS
Octadecanol	< 0.05	PSS – BD; PSS – RAS
Malic acid	< 0.05	PSS – BD; PSS – RAS
Urea	< 0.05	PSS – BD; PSS – RAS
Linoleic acid	< 0.05	PSS – BD; RAS – BD
Pyroglutamic acid	< 0.05	PSS – BD; RAS – BD
Tetradecanoic acid	< 0.05	PSS – BD; RAS – BD
Melezitose	< 0.05	PSS – BD; RAS – BD
Phosphoric acid	< 0.05	PSS – BD; RAS – BD
Eicosanoic acid	< 0.05	PSS – BD; RAS – BD
Ornithine	< 0.05	PSS – BD; RAS – BD
L-Proline	< 0.05	PSS – BD; RAS – BD; PSS – RAS
L-Serine	< 0.05	PSS – BD; RAS – BD; PSS – RAS
Triethanolamine	< 0.05	PSS – BD; RAS – BD; PSS – RAS
L-Leucine	< 0.05	PSS – BD; RAS – BD; PSS – RAS
Fructose	< 0.05	PSS – BD; RAS – BD; PSS – RAS
Glutaric acid	< 0.05	PSS – BD; RAS – BD; PSS – RAS
Succinic acid	< 0.05	PSS – BD; RAS – BD; PSS – RAS
Glucopyranose	< 0.05	PSS – BD; RAS – BD; RAS – PSS
Lactic acid	< 0.05	PSS – BD; RAS – BD; RAS – PSS
Oleic acid	< 0.05	PSS – BD; RAS – BD; RAS – PSS
Uridine	< 0.05	PSS – BD; RAS – BD; RAS – PSS
Suberic acid	< 0.05	PSS – BD; RAS – BD; RAS – PSS
Sorbitol	< 0.05	PSS – BD; RAS – BD; RAS – PSS
L-valine	< 0.05	PSS – BD; RAS – BD; RAS – PSS
Maltose	< 0.05	PSS – RAS
Octadecanoic acid	< 0.05	RAS – BD; RAS – PSS
4-Hydroxybenzoic acid	< 0.05	RAS – BD; RAS – PSS
Glycerol 3-phosphate	< 0.05	RAS – BD; RAS – PSS
Cysteine	< 0.05	RAS – BD; RAS – PSS

## Discussion

This study demonstrated that untargeted salivary metabolomic profiling can effectively differentiate BD, PSS, and RAS, which share similar oral manifestations but distinct systemic backgrounds. Using GC/MS and multivariate analyses, we identified unique metabolic signatures in each condition, with seven metabolites—malonic acid, aspartic acid, pyroglutamic acid, L-proline, sorbitol, decanoic acid, and hexadecanoic acid—showing high variable-importance scores. Among these, malonic and aspartic acids were markedly elevated in BD, suggesting disease-specific alterations in oxidative and amino-acid metabolism [36]. These saliva-based patterns parallel our previous metabolomics studies in BD, where supervised models clearly separated BD from comparators and yielded compact biomarker panels in serum and urine, as well as disease-specific synovial signatures in BD arthritis [37–39]. Collectively, these results underscore the potential of salivary metabolomics as a non-invasive diagnostic approach reflecting systemic immune–metabolic remodeling.

The BD-dominant rise in malonic and aspartic acids indicates a shift toward glycolytic and anaplerotic metabolism, a recognized feature of activated immune and endothelial cells [40]. Malonic acid, a competitive inhibitor of succinate dehydrogenase, can promote succinate accumulation and ROS generation, triggering HIF-1α-driven IL-1β production. This pro-inflammatory metabolic loop mirrors findings from serum metabolomics, where a five-marker panel enriched for medium-chain fatty acids and monosaccharides (decanoic acid and fructose ↑ , linoleic and oleic acids ↓) achieved near-perfect discrimination (AUC ≈ 1.0) [39]. Complementary urine profiling identified a ten-metabolite panel involv-ing purine and carbohydrate fluxes (guanine, mannose, L-citrulline), indicating disturbed energy and nitrogen metabolism [38]. Our salivary results thus converge with cross-biofluid evidence that BD is characterized by dysregulated fatty-acid oxidation and carbohydrate–purine turnover. In addition, the elevated aspartic acid may reflect enhanced transamination between aspartate and glutamate, fueling nucleotide synthesis and cell proliferation. These alterations resemble metabolic fingerprints observed in rheumatoid arthritis synovial fluid [37], suggesting that BD shares a core inflammatory metabolic phenotype centered on mitochondrial stress and amino-acid reprogramming. Cross-matrix convergence in oxidative pathways is particularly notable. Methionine sulfoxide and citrulline were elevated in BD synovial fluid, linking to neutrophil-driven oxidative stress. Serum signatures rich in medium-chain fatty acids and keto-hexoses like-wise indicate redox-coupled endothelial–immune reprogramming. The concurrent detection of malonic acid and other redox-active metabolites in saliva reinforces the idea that oxidative stress is a shared mechanistic axis across BD matrices, spanning circulation. Such consistency across biofluids underscores the systemic nature of metabolic inflammation in BD.

In PSS, the predominant increases in glycine, urea, glucose, and octadecanol reflect adaptive responses to glandular dysfunction and oxidative stress. Glycine serves as a glutathione precursor, and elevated urea indicates heightened nitrogen turnover via the arginine–proline cycle [41]. These changes align with prior urinary and plasma metabolomics of PSS highlighting disturbed amino-acid and redox metabolism [42]. Our patten is consistent with a recent report demonstrating higher levels of energy- and redox-linked metabolites (lactate, alanine, malate) together with T-cell growth amino acids (arginine, leu-cine, isoleucine, valine) in PSS [43].

Contrasting with PSS, BD shows stronger remodeling of fatty-acid and carbohydrate–purine metabolism across serum and urine, mirroring the salivary shifts reported here. Together these findings support the concept that salivary metabolites mirror systemic re-dox and osmotic adaptation in PSS but discriminate it clearly from the oxidative fatty-acid phenotype dominant in BD.

RAS exhibited higher levels of octadecanoic acid, 4-hydroxybenzoic acid, glycer-ol-3-phosphate, and cysteine. These metabolites are linked to lipid remodeling and anti-oxidant defense. Elevated cysteine likely supports glutathione synthesis and epithelial repair [44], whereas increased glycerol-3-phosphate and long-chain fatty acids could be involved in active membrane turnover. Consistent with recent LC–MS metabolomics integrating 16S rRNA sequencing, RAS saliva showed coordinated dysregulation in amino acid, glycerophospholipid, and glutathione metabolism, together with selective accumulation of lipid-like molecules [45]. These metabolic shifts were tightly correlated with oral microbiota changes, suggesting that microbial–lipid interactions may drive epithelial injury and repair dynamics in RAS [46].

Our findings support saliva as a practical matrix for early diagnostic triage when oral dryness and mucosal symptoms precede definitive serology or biopsy. Integrating these multi-matrix signatures into multivariate models could assist in early stratification of patients presenting with nonspecific oral ulcers and differentiate those at risk for systemic BD or PSS.

Several limitations should be acknowledged. The sample size was small and single-center, and clinical heterogeneity including variation in disease duration and symptom severity or medication effects could not be fully controlled due to the exploratory design and limited cohort size. Because both previous BD biofluid studies and the present saliva work employed gas chromatography/time-of-flight mass spectrometry (GC/TOF-MS), orthogonal validation with liquid chromatography–tandem mass spectrometry (LC–MS/MS) and nuclear magnetic resonance spectroscopy (NMR) is warranted to confirm metabolite identities and enhance quantitation across shared pathways. In this regard, this preliminary study can be regarded as having achieved its intended objectives to assess the potential candidate markers of saliva sample. In future studies, we will perform detailed clinical subgrouping and treatment-status information to minimize confounding effects, while integrating metabolomic, proteomic, and transcriptomic datasets to elucidate the network linking local mucosal metabolism to systemic autoimmunity and validate candidate biomarkers in multi-center cohorts.

## Conclusions

In conclusion, this integrated analysis provides the first comparative salivary metabolomic profile of BD, PSS, and RAS and positions it within a cross-biofluid framework established by our previous serum, urine, and synovial fluid studies. Elevated malonic and aspartic acids in BD indicate mitochondrial and amino-acid dysregulation; in PSS, in-creased glycine and urea reflect redox and osmotic adaptation; and in RAS, lipid and cysteine enrichment signifies localized repair. Across matrices, BD shows a consistent metabolic triad of fatty-acid remodeling, carbohydrate–purine flux, and oxidative amino-acid stress. This study therefore positions salivary metabolomics as a practical, non-invasive platform for the differentiation of oral ulcerative diseases along the autoimmune continuum.
