# Supplementary material for: Metabolite profiling of saliva for the discrimination of Behcet’s disease, Sjögren’s syndrome, and recurrent aphthous stomatitis
Source: PLoS One. 2026 Jun 9;21(6):e0351403. doi: 10.1371/journal.pone.0351403 (PMC13249169; doi:10.1371/journal.pone.0351403)
Supplement: Attachment — Submitted filename: Response_PLOS ONE_0403.docx [file pone.0351403.s002.docx]

**Responses to Reviewersʼ Comments**

**Manuscript:** PONE-D-25-64353

**Title:** Metabolite profiling of saliva for the discrimination of Behcet’s disease, Sjögren’s syndrome, and recurrent aphthous stomatitis

**Corresponding author:** Jiwon Hwang

We appreciate the editors and reviewers of *‘PLOS ONE’* for taking the time to review our study. We also sincerely thank you for your constructive comments. The authors have carefully considered all comments provided by the reviewers and the editor, and we deeply appreciate the insightful suggestions. A revised manuscript has been prepared accordingly, and all changes have been clearly indicated in red font throughout the manuscript for ease of reference.

We respectfully submit the revised version for reconsideration for publication in *‘PLOS ONE’.* Our detailed responses to each comment are provided below.

**<Reviewer 1>**

**Comment 1:**

I. Introduction and Abstract: The research question and key findings were clearly mentioned in the Abstract. The research aim and novelty of this study in the context of existing literature and research that has been done already have been made very clear however, some studies whose findings were given had no references. The gap in the literature was mentioned however, some were not referenced.

***Response:*** Thank you for this helpful comment. We agree that several statements describing previous studies and the existing gap in the literature were insufficiently referenced. We have carefully revised the Introduction and added appropriate references to support statements regarding prior findings, the current state of salivary metabolomics research, and the specific gap addressed by our study. We also refined the description of the study novelty to make the distinction between existing evidence and the remaining unmet need clearer.

**Comment 2:**

II. Materials and Methods

The experiment and technology employed appears to be technically sound and appropriate for the research objective. Data was collected and interpreted appropriately with conformation to ethical guidelines. Procedural information has been provided to allow for reproducibility. The technology employed was appropriate for metabolomics.

However, it is not clearly stated how the authors arrived at the sample size and the procedural techniques employed were not referenced. If these techniques are new or modified, it was not stated and no reason was given as well. I must say that I am not an expert in the experiments and technical procedures employed in the study.

***Response:*** Thank you for your comment. To address the reviewer’s concern regarding the sample size, we benchmarked our study design against recent pilot metabolomics studies on previous studies. For instance, a metabolomics study on Behcet’s disease (BD) utilized 20 patients to successfully identify sweat biomarkers, including L-pyroglutamic acid-a key metabolite also identified in our study [1]. Similarly, a study on primary Sjögren’s syndrome (pSS) employed a cohort of 32 patients to establish salivary metabolic profiles [2]. Our total cohort of 43 patients is well within the range of these published exploratory studies, providing sufficient statistical power to yield clear discrimination in our PCA and PLS-DA models.

In addition, we have revised the Materials and Methods section to include appropriate references for the experimental procedures. We have also clarified that the methods used in this study are based on established protocols with minor modifications.

**[Revision: lines 101]** Sample preparation was performed by modifying a previously established protocol.

1.	Cui XX, Zhang LM, Su GN, Kijlstra A, Yang PZ. Specific sweat metabolite profile in ocular Behcet's disease. Int Immunopharmacol. 2021;97. doi: 10.1016/j.intimp.2021.107812. PubMed PMID: WOS:000679152100006.

2.	Li Z, Mu Y, Guo CL, You X, Liu XY, Li Q, et al. Analysis of the saliva metabolic signature in patients with primary Sjogren's syndrome. Plos One. 2022;17(6). doi: 10.1371/journal.pone.0269275. PubMed PMID: WOS:000843567600028.

**Comment 3:**

I. Results and Discussion

The results support the conclusions provided and the authors discussed the limitations of the study. Tables and figures are clear and readable, with accurate captions. Figures and tables do support findings and presentation of results was appropriate for the data type being presented.

However, axes were not labelled completely in Figures 2 and 3.

***Response:*** Thank you for your helpful comment. We have revised Figures 2 and 3 by adding the missing axis labels. In Figure 2, 'Samples' has been added as the x-axis label and 'Metabolites' as the y-axis label. In Figure 3, 'Metabolites' has been added as the y-axis label, and the group panel heading has been clarified as 'Group.’

**Comment 4:**

Other points/comments to the editor.

1. Statistical analysis;

I am not an expert in the field of statistical analysis however, to the best of my knowledge, the statistical tests employed were an appropriate fit to help arrive at the conclusions as mentioned in the article.

***Response:*** We appreciate the reviewer’s comment regarding the appropriateness of the statistical analysis.

**Comment 5:**

The authors made no statement about the availability of the data underlying the findings described in the manuscript.

***Response:*** Thank you for this important comment. We agree that the availability of the data underlying the findings was not clearly stated in the original submission. We have now added a Data Availability Statement in the revised manuscript in accordance with the PLOS ONE data policy. Because the study involves de-identified human participant data, the dataset cannot be shared publicly. We have clarified that requests for access to the minimal de-identified dataset should be directed to the appropriate institutional body of the original IRB-approving institution, subject to ethical and legal requirements.

**[Revision: lines 302-307]** Data Availability: The data underlying the findings of this study contain de-identified human participant data and cannot be shared publicly due to ethical and legal considerations related to participant confidentiality. Requests for access to the minimal de-identified dataset may be directed to the Institutional Review Board of Samsung Changwon Hospital, subject to institutional review and applicable regulations.

**<Reviewer 2>**

Materials and Methods

**Comment 1:** In line 74, where it states, "The study subjects include patients with RAS, BD, and PSS," it is necessary to specify how the diagnoses of these entities were carried out.

***Response:*** Thank you for this important comment. We agree that the diagnostic definitions for each study group should be stated explicitly. We have revised the Materials and Methods section accordingly.

**[Revision: lines 78-84]** All diagnoses were established by board-certified rheumatologists. BD was defined according to the Japanese diagnostic criteria for Behcet’s disease [22], and PSS was classified according to the 2016 American College of Rheumatology (ACR)/European League Against Rheumatism (EULAR) classification criteria for primary Sjögren's syndrome [23]. RAS was defined as recurrent oral ulceration without systemic features suggestive of BD or PSS and without fulfillment of corresponding diagnostic or classification criteria.

**Comment 2:** Describing the sample type, handling, and evaluation for the ANA and Anti-Ro tests is necessary given their significance in the results.

***Response:*** Thank you for this important comment. We agree that the methodological details for ANA and anti-Ro testing should be described more explicitly because these variables were relevant to the interpretation of the results. We have revised the Materials and Methods section for the relevant sentences.

**[Revision: lines 86-89]** Antinuclear antibody (ANA) and anti-Ro antibody results were obtained from routine clinical laboratory testing in electronic medical records. Both tests were performed on serum samples, and positivity was determined according to the reference ranges and cut-off values used by the institutional laboratory at the time of testing.

Results

**Comment 1:** Since the results of interest in Table 1 focus on the Antinuclear Antibody Test (ANA) values, it is necessary to briefly explain how these results are interpreted to suggest the presence versus the possibility of an autoimmune disease.

***Response:*** Thank you for this helpful comment. We agree that the ANA results shown in Table 1 require a brief interpretive context. We have revised the Results section to clarify that ANA was treated as a descriptive screening variable for autoimmune diseases, not as a classification item for PSS. We also noted that all PSS patients were positive for anti-Ro antibodies.

**[Revision: lines 138-141]** Although ANA is not included in the 2016 ACR/EULAR classification criteria for PSS, it is a basic screening test for autoimmune disease. In contrast, all PSS patients were positive for anti-Ro antibodies, consistent with the serologic item directly relevant to the classification criteria.

**Comment 2:** In paragraphs 150-156, the authors suggest that, based on their results, some metabolites are present in higher concentrations in patients with PSS compared to RAS and BD. They then present other metabolites that also show higher concentrations in PSS compared to RAS and BD. It would be necessary to explain why these are presented separately and not together, given that they refer to the same disease. Lines 154-156 suggest differences in metabolite levels but do not explain which specific metabolites are observed to be differentially expressed to distinguish between diseases, as presented in Figure 3.

***Response:*** Thank you for your insightful comment. We agree that the original text was unnecessarily divided into two separate lists. We have merged these into a single description and revised the text to clarify the relationship between the metabolites identified in Figures 2 and 3.

**[Revision: lines 166-173]** As shown in Fig 2, it can be observed that the content of urea, fructose, succinic acid, malic acid, glucose, glycine, triethanolamine, L-serine, L-leucine, octadecanol, glutaric acid, L-proline, putrescine, nonanoic acid, dodecanoic acid, 3-hydroxypropanoic acid, cholesterol, aspartic acid, and myo-inositol is higher in PSS compared to RAS and BD. These findings indicate significant inter-disease differences in salivary metabolite levels. Notably, among these metabolites, aspartic acid and L-proline were also identified as key discriminatory metabolites in the PLS-DA analysis (Fig. 3), further supporting their potential as biomarkers for discriminating between RAS, BD, and PSS.

**Comment 3:** In paragraphs 158-161, it will be necessary to explain in more detail the results presented in Table 4, allowing for a clear understanding of the differences between diseases.

***Response:*** Thank you for your comment. We would like to clarify that the detailed differences in metabolite levels among BD, PSS, and RAS based on Table 4 are already described in lines 178-183, where disease-specific elevations are specified for each group. We hope this adequately addresses your concern.

**Comment 4:** Develop the suggestion presented in lines 174-175, which indicates disease-dependent specific salivary metabolites.

***Response:*** Thank you for this helpful suggestion. We have revised the corresponding sentence to more clearly describe disease-dependent salivary metabolites by specifying representative metabolites associated with BD, PSS, and RAS.

**[Revision: lines 190-193]** Collectively, these results indicate that specific salivary metabolites exhibit disease-dependent changes, with malonic acid and aspartic acid elevated in BD, L-proline increased in PSS, and cysteine elevated in RAS, suggesting their potential as non-invasive biomarkers for discriminating among RAS, BD, and PSS.

**Comment 5:** In lines 176-177, explain the differences in the regulation of metabolic pathways for each disease that support the discovery versus previous publications.

***Response:*** Thank you for this insightful comment. While the Discussion already addressed metabolite-related biological functions, we have revised the corresponding section to more clearly emphasize differences in metabolic pathway regulation.

**[Revision: lines 195-198]** In particular, malonic acid and aspartic acid in BD are linked to oxidative stress and immune regulation [26-28], and putrescine and L-proline in PSS reflect anti-inflammatory responses and tissue repair [29-32].

**Comment 6:** Develop the presentation suggestion in lines 178-179.

***Response:*** Thank you for this helpful suggestion. We have revised the corresponding sentence to provide a more explicit description of the potential clinical application of salivary metabolomics, particularly in the early differentiation of patients with nonspecific oral symptoms.

**[Revision: lines 200-203]** It may be particularly helpful in the early differentiation of patients presenting with nonspecific oral ulcers, where clinical symptoms overlap among BD, PSS, and RAS, potentially supporting timely diagnosis and reducing the need for invasive procedures such as biopsy.

Thank you again for your time, consideration, and constructive feedback. We hope that the revisions have adequately addressed all concerns, and we respectfully await your further consideration.

Sincerely,

Jiwon Hwang, MD.PhD.

On behalf of all authors

Associate Professor

Division of Rheumatology, Department of Internal Medicine

Kyung Hee University Hospital, Kyung Hee University College of Medicine

Seoul 02447

South Korea

Tel: +82-2-958-8200

Fax: +82-2-968-1848

E-mail: [ninedw@empas.com](mailto:ninedw@empas.com)
