# Supplementary material for: Metabolite profiling of saliva for the discrimination of Behcet’s disease, Sjögren’s syndrome, and recurrent aphthous stomatitis
Source: PLoS One. 2026 Jun 9;21(6):e0351403. doi: 10.1371/journal.pone.0351403 (PMC13249169; doi:10.1371/journal.pone.0351403)
Supplement: Attachment — Submitted filename: Response to Reviewers_PONE-D-25-64353R1.docx [file pone.0351403.s003.docx]

**Responses to Reviewersʼ Comments**

**Manuscript:** PONE-D-25-64353R1

**Title:** Metabolite profiling of saliva for the discrimination of Behcet’s disease, Sjögren’s syndrome, and recurrent aphthous stomatitis

**Corresponding author:** Jiwon Hwang

We would like to express our sincere gratitude to the editor and reviewers of ‘*PLOS ONE*’ for their continued evaluation of our manuscript and for the constructive comments provided during this second round of review. We have carefully addressed the remaining issues raised by the reviewers and revised the manuscript accordingly. All changes have been clearly indicated in red font throughout the revised manuscript. We respectfully resubmit the revised version for reconsideration for publication in *‘PLOS ONE’.* Our detailed responses to each comment are provided below.

**<Reviewer 1: Methods Section>**

**Comment 1:**

Justifying sample size based on precedence from prior comparable studies can be scientifically acceptable, but it is generally considered a pragmatic or feasibility-based justification, and not the strongest form of statistical justification in rare diseases and exploratory metabolomics research.

This study revolves around 3 autoimmune diseases which are not common. There is however, not much statistical information on the prevalence of diseases to objectively prove their rarity, which could be a valid reason for justification of sample size based on prior comparable studies. Authors should please address this and provide what is known in the literature about the prevalence and rarity of these conditions objectively.

Authors should also explicitly indicate whether the study is an exploratory one or a confirmatory one. A clear indication that the study is an exploratory one or a hypothesis-generating one can provide a stronger scientific justification for the pragmatic determination of the sample size based on previous published metabolomics studies, because using previous studies alone is not equivalent to formal sample size calculation and does not provide adequate statistical power.

The study, being an exploratory one, has been clearly mentioned in the discussion, with the limitation on the small sample size clearly acknowledged. However, the type of study and the justification for the sample size should be clearly detailed in the methods section.

***Response:*** Thank you for this important comment. We agree that determining the sample size based on previous comparable studies represents a pragmatic and feasibility-based rationale rather than a formal statistical power calculation. In response, we have revised the Methods section to explicitly state the relevant contents with appropriate references.

**[Revision: lines 100-109]** This study was designed as an exploratory, hypothesis-generating salivary metabolomics study rather than a confirmatory study. BD and PSS are relatively uncommon systemic immune-mediated diseases, whereas RAS is more common but clinically heterogeneous and was included as a disease comparator because of its overlapping oral manifestations. Given this exploratory design, a formal sample size calculation based on a prespecified effect size and statistical power was not performed. Instead, the sample size was determined pragmatically by considering the epidemiological characteristics of the study diseases, the feasibility of recruiting well-characterized participants, the availability of saliva samples suitable for GC/MS-based metabolomic profiling, and sample sizes used in previous comparable metabolomics studies [24-26].

24.	Kageyama G, Saegusa J, Irino Y, Tanaka S, Tsuda K, Takahashi S, et al. Metabolomics analysis of saliva from patients with primary Sjogren's syndrome. Clinical and Experimental Immunology. 2015;182(2):149-53. doi: 10.1111/cei.12683. PubMed PMID: WOS:000362955000005.

25.	Li Z, Mu Y, Guo CL, You X, Liu XY, Li Q, et al. Analysis of the saliva metabolic signature in patients with primary Sjogren's syndrome. Plos One. 2022;17(6). doi: 10.1371/journal.pone.0269275. PubMed PMID: WOS:000843567600028.

26.	Alrashdan MS, Alnemri D, Al-Qarqaz FA, Alawneh KM, Al-Rawi NH, Bouzid A, et al. Characterization of potential salivary biomarkers in Behçet's disease. Internal and Emergency Medicine. 2025;20(2):413-21. doi: 10.1007/s11739-024-03801-6. PubMed PMID: WOS:001346258100002.

**<Reviewer 2>**

**Comment 1:** All comments have been properly addressed and clarified in the work "Metabolite profiling of saliva for the discrimination of Behcet’s disease, Sjögren’s syndrome, and recurrent aphthous stomatitis"

***Response:*** We thank the reviewer for the positive comment and for confirming that all previous concerns have been adequately addressed.

Thank you again for your time, consideration, and constructive feedback. We hope that the revisions have adequately addressed all concerns, and we respectfully await your further consideration.

Sincerely,

Jiwon Hwang, MD.PhD.

On behalf of all authors

Associate Professor

Division of Rheumatology, Department of Internal Medicine

Kyung Hee University Hospital, Kyung Hee University College of Medicine

Seoul 02447

South Korea

Tel: +82-2-958-8200

Fax: +82-2-968-1848

E-mail: [ninedw@empas.com](mailto:ninedw@empas.com)
