# Supplementary material for: Metabolite profiling of saliva for the discrimination of Behcet’s disease, Sjögren’s syndrome, and recurrent aphthous stomatitis
Source: PLoS One. 2026 Jun 9;21(6):e0351403. doi: 10.1371/journal.pone.0351403 (PMC13249169; doi:10.1371/journal.pone.0351403)
Supplement: Peer review history

# Peer review: Decision Letter 0

**DOI:** 10.1371/journal.pone.0351403.r001

**Authors:** Ronell Bologna-Molina

11 Mar 2026

PONE-D-25-64353Metabolite profiling of saliva for the discrimination of Behcet’s disease, Sjögren’s syndrome, and recurrent aphthous stomatitisPLOS One

Dear Dr. Kim,

Thank you for submitting your manuscript to PLOS ONE. After careful consideration, we feel that it has merit but does not fully meet PLOS ONE’s publication criteria as it currently stands. Therefore, we invite you to submit a revised version of the manuscript that addresses the points raised during the review process.

Please perform all the changes and suggetions made by the reviewers.

Please submit your revised manuscript by Apr 25 2026 11:59PM. If you will need more time than this to complete your revisions, please reply to this message or contact the journal office at plosone@plos.org. When you're ready to submit your revision, log on to https://www.editorialmanager.com/pone/ and select the 'Submissions Needing Revision' folder to locate your manuscript file.

Please include the following items when submitting your revised manuscript:

- A letter that responds to each point raised by the academic editor and reviewer(s). You should upload this letter as a separate file labeled 'Response to Reviewers'.
- A marked-up copy of your manuscript that highlights changes made to the original version. You should upload this as a separate file labeled 'Revised Manuscript with Track Changes'.
- An unmarked version of your revised paper without tracked changes. You should upload this as a separate file labeled 'Manuscript'.

If you would like to make changes to your financial disclosure, please include your updated statement in your cover letter. Guidelines for resubmitting your figure files are available below the reviewer comments at the end of this letter.

If applicable, we recommend that you deposit your laboratory protocols in protocols.io to enhance the reproducibility of your results. Protocols.io assigns your protocol its own identifier (DOI) so that it can be cited independently in the future. For instructions see: https://journals.plos.org/plosone/s/submission-guidelines#loc-laboratory-protocols. Additionally, PLOS ONE offers an option for publishing peer-reviewed Lab Protocol articles, which describe protocols hosted on protocols.io. Read more information on sharing protocols at https://plos.org/protocols?utm_medium=editorial-email&utm_source=authorletters&utm_campaign=protocols.

We look forward to receiving your revised manuscript.

Kind regards,

Ronell Bologna-Molina

Academic Editor

PLOS One

Journal Requirements:

When submitting your revision, we need you to address these additional requirements.

1. Please ensure that your manuscript meets PLOS ONE's style requirements, including those for file naming. The PLOS ONE style templates can be found at

https://journals.plos.org/plosone/s/file?id=wjVg/PLOSOne_formatting_sample_main_body.pdf and https://journals.plos.org/plosone/s/file?id=ba62/PLOSOne_formatting_sample_title_authors_affiliations.pdf

2. We note that your ethical committee requested corrections made to your application. Please could you provide the ethical approval document, confirming that full approval was given?

3. Your ethics statement should only appear in the Methods section of your manuscript. If your ethics statement is written in any section besides the Methods, please delete it from any other section.

4. Thank you for stating the following in the Acknowledgments Section of your manuscript:

This work was supported by the Sungkyunkwan University School of Medicine Samsung Changwon Hospital, and by the Regional Innovation System & Education (RISE) program through the Jeonbuk RISE Center, funded by the Ministry of Education (MOE) and the Jeonbuk State, Republic of Korea (2025-RISE-13-JJU).

We note that you have provided funding information that is not currently declared in your Funding Statement. However, funding information should not appear in the Acknowledgments section or other areas of your manuscript. We will only publish funding information present in the Funding Statement section of the online submission form.

Please remove any funding-related text from the manuscript and let us know how you would like to update your Funding Statement. Currently, your Funding Statement reads as follows:

This work was supported by the Sungkyunkwan University School of Medicine, Samsung Changwon Hospital.

Recipient: J.H

This work was also supported by the Regional Innovation System & Education (RISE) Program through the Jeonbuk RISE Center, funded by the Ministry of Education (MOE) and the Jeonbuk State, Republic of Korea.

Grant number: 2025-RISE-13-JJU

Recipient: S.K.

Please include your amended statements within your cover letter; we will change the online submission form on your behalf.

5. If the reviewer comments include a recommendation to cite specific previously published works, please review and evaluate these publications to determine whether they are relevant and should be cited. There is no requirement to cite these works unless the editor has indicated otherwise.

[Note: HTML markup is below. Please do not edit.]

Reviewers' comments:

Reviewer's Responses to Questions

**Comments to the Author**

1. Is the manuscript technically sound, and do the data support the conclusions?

The manuscript must describe a technically sound piece of scientific research with data that supports the conclusions. Experiments must have been conducted rigorously, with appropriate controls, replication, and sample sizes. The conclusions must be drawn appropriately based on the data presented.

Reviewer #1: Partly

Reviewer #2: Yes

**********

2. Has the statistical analysis been performed appropriately and rigorously?

Reviewer #1: Yes

Reviewer #2: Yes

**********

3. Have the authors made all data underlying the findings in their manuscript fully available?

The PLOS Data policy requires authors to make all data underlying the findings described in their manuscript fully available without restriction, with rare exception (please refer to the Data Availability Statement in the manuscript PDF file). The data should be provided as part of the manuscript or its supporting information, or deposited to a public repository. For example, in addition to summary statistics, the data points behind means, medians and variance measures should be available. If there are restrictions on publicly sharing data—e.g. participant privacy or use of data from a third party—those must be specified.

Reviewer #1: No

Reviewer #2: Yes

**********

4. Is the manuscript presented in an intelligible fashion and written in standard English?

PLOS ONE does not copyedit accepted manuscripts, so the language in submitted articles must be clear, correct, and unambiguous. Any typographical or grammatical errors should be corrected at revision, so please note any specific errors here.

Reviewer #1: Yes

Reviewer #2: Yes

**********

5. Review Comments to the Author

Please use the space provided to explain your answers to the questions above. You may also include additional comments for the author, including concerns about dual publication, research ethics, or publication ethics. (Please upload your review as an attachment if it exceeds 20,000 characters)

Reviewer #1: A. Summary of research and overall impression:

This study investigated the use of salivary metabolomics as a practical, non-invasive platform for differentiating oral ulcerative diseases within the spectrum of inflammatory and autoimmune diseases. The study results indicated that specific salivary metabolites exhibit significant disease-dependent changes and can be used as potential non-invasive biomarkers for discriminating recurrent aphthous ulcers (RAS), Behcet’s disease (BD), and Sjogren’s syndrome (PSS). The authors demonstrated good scientific writing and technical rigour with conclusions that were consistent with the results obtained. I therefore recommend that this research article be accepted for publication after the major and minor issues identified below have been addressed.

B. Discussion of specific areas for improvement

1. Major Issues:

I. Introduction and Abstract

The research question and key findings were clearly mentioned in the Abstract.

The research aim and novelty of this study in the context of existing literature and research that has been done already have been made very clear however, some studies whose findings were given had no references.

The gap in the literature was mentioned however, some were not referenced .

II. Materials and Methods

The experiment and technology employed appears to be technically sound and appropriate for the research objective. Data was collected and interpreted appropriately with conformation to ethical guidelines. Procedural information has been provided to allow for reproducibility. The technology employed was appropriate for metabolomics.

However, it is not clearly stated how the authors arrived at the sample size and the procedural techniques employed were not referenced. If these techniques are new or modified, it was not stated and no reason was given as well. I must say that I am not an expert in the experiments and technical procedures employed in the study.

2. Minor Issues:

I. Results and Discussion

The results support the conclusions provided and the authors discussed the limitations of the study. Tables and figures are clear and readable, with accurate captions. Figures and tables do support findings and presentation of results was appropriate for the data type being presented.

However, axes were not labelled completely in Figures 2 and 3.

C. Other points/comments to the editor.

1. Statistical analysis;

I am not an expert in the field of statistical analysis however, to the best of my knowledge, the statistical tests employed were an appropriate fit to help arrive at the conclusions as mentioned in the article.

2. The authors made no statement about the availability of the data underlying the findings described in the manuscript.

Reviewer #2: Salivary Metabolite Profiling for the Discrimination of Behcet's Disease and Sjögren's Syndrome

First, I appreciate the opportunity to review such an interesting work.

Materials and Methods

1. In line 74, where it states, "The study subjects include patients with RAS, BD, and PSS," it is necessary to specify how the diagnoses of these entities were carried out.

2. Describing the sample type, handling, and evaluation for the ANA and Anti-Ro tests is necessary given their significance in the results.

Results

1. Since the results of interest in Table 1 focus on the Antinuclear Antibody Test (ANA) values, it is necessary to briefly explain how these results are interpreted to suggest the presence versus the possibility of an autoimmune disease.

2. In paragraphs 150-156, the authors suggest that, based on their results, some metabolites are present in higher concentrations in patients with PSS compared to RAS and BD. They then present other metabolites that also show higher concentrations in PSS compared to RAS and BD. It would be necessary to explain why these are presented separately and not together, given that they refer to the same disease. Lines 154-156 suggest differences in metabolite levels but do not explain which specific metabolites are observed to be differentially expressed to distinguish between diseases, as presented in Figure 3.

3. In paragraphs 158-161, it will be necessary to explain in more detail the results presented in Table 4, allowing for a clear understanding of the differences between diseases.

4. Develop the suggestion presented in lines 174-175, which indicates disease-dependent specific salivary metabolites.

5. In lines 176-177, explain the differences in the regulation of metabolic pathways for each disease that support the discovery versus previous publications.

6. Develop the presentation suggestion in lines 178-179.

Overall, the development of the Discussion and Conclusion is very appropriate and interesting.

**********

6. PLOS authors have the option to publish the peer review history of their article (what does this mean?). If published, this will include your full peer review and any attached files.

If you choose “no”, your identity will remain anonymous but your review may still be made public.

**Do you want your identity to be public for this peer review?** For information about this choice, including consent withdrawal, please see our Privacy Policy.

Reviewer #1: No

Reviewer #2: No

**********

[NOTE: If reviewer comments were submitted as an attachment file, they will be attached to this email and accessible via the submission site. Please log into your account, locate the manuscript record, and check for the action link "View Attachments". If this link does not appear, there are no attachment files.]

To ensure your figures meet our technical requirements, please review our figure guidelines: https://journals.plos.org/plosone/s/figures

You may also use PLOS’s free figure tool, NAAS, to help you prepare publication quality figures: https://journals.plos.org/plosone/s/figures#loc-tools-for-figure-preparation.

NAAS will assess whether your figures meet our technical requirements by comparing each figure against our figure specifications.

# Author comment: Author response to Decision Letter 1

**DOI:** 10.1371/journal.pone.0351403.r002

3 Apr 2026

Responses to Reviewersʼ Comments

Manuscript: PONE-D-25-64353

Title: Metabolite profiling of saliva for the discrimination of Behcet’s disease, Sjögren’s syndrome, and recurrent aphthous stomatitis

Corresponding author: Sooah Kim

We appreciate the editors and reviewers of ‘PLOS ONE’ for taking the time to review our study. We also sincerely thank you for your prompt and constructive comments. The authors have carefully considered all comments provided by the reviewers and the editor, and we deeply appreciate the insightful suggestions. A revised manuscript has been prepared accordingly, and all changes have been clearly indicated in red font throughout the manuscript for ease of reference.

We respectfully submit the revised version for reconsideration for publication in ‘PLOS ONE’. Our detailed responses to each comment are provided below.

<Reviewer 1>

Comment 1:

I. Introduction and Abstract: The research question and key findings were clearly mentioned in the Abstract. The research aim and novelty of this study in the context of existing literature and research that has been done already have been made very clear however, some studies whose findings were given had no references. The gap in the literature was mentioned however, some were not referenced.

Response: Thank you for this helpful comment. We agree that several statements describing previous studies and the existing gap in the literature were insufficiently referenced. We have carefully revised the Introduction and added appropriate references to support statements regarding prior findings, the current state of salivary metabolomics research, and the specific gap addressed by our study. We also refined the description of the study novelty to make the distinction between existing evidence and the remaining unmet need clearer.

Comment 2:

II. Materials and Methods

The experiment and technology employed appears to be technically sound and appropriate for the research objective. Data was collected and interpreted appropriately with conformation to ethical guidelines. Procedural information has been provided to allow for reproducibility. The technology employed was appropriate for metabolomics.

However, it is not clearly stated how the authors arrived at the sample size and the procedural techniques employed were not referenced. If these techniques are new or modified, it was not stated and no reason was given as well. I must say that I am not an expert in the experiments and technical procedures employed in the study.

Response: Thank you for your comment. To address the reviewer’s concern regarding the sample size, we benchmarked our study design against recent pilot metabolomics studies on previous studies. For instance, a metabolomics study on Behcet’s disease (BD) utilized 20 patients to successfully identify sweat biomarkers, including L-pyroglutamic acid-a key metabolite also identified in our study [1]. Similarly, a study on primary Sjögren’s syndrome (pSS) employed a cohort of 32 patients to establish salivary metabolic profiles [2]. Our total cohort of 43 patients is well within the range of these published exploratory studies, providing sufficient statistical power to yield clear discrimination in our PCA and PLS-DA models.

In addition, we have revised the Materials and Methods section to include appropriate references for the experimental procedures. We have also clarified that the methods used in this study are based on established protocols with minor modifications.

[Revision: lines 101] Sample preparation was performed by modifying a previously established protocol.

Comment 3:

I. Results and Discussion

The results support the conclusions provided and the authors discussed the limitations of the study. Tables and figures are clear and readable, with accurate captions. Figures and tables do support findings and presentation of results was appropriate for the data type being presented.

However, axes were not labelled completely in Figures 2 and 3.

Response: Thank you for your helpful comment. We have revised Figures 2 and 3 by adding the missing axis labels. In Figure 2, 'Samples' has been added as the x-axis label and 'Metabolites' as the y-axis label. In Figure 3, 'Metabolites' has been added as the y-axis label, and the group panel heading has been clarified as 'Group.’

Comment 4:

Other points/comments to the editor.

1. Statistical analysis;

I am not an expert in the field of statistical analysis however, to the best of my knowledge, the statistical tests employed were an appropriate fit to help arrive at the conclusions as mentioned in the article.

Response: We appreciate the reviewer’s comment regarding the appropriateness of the statistical analysis.

Comment 5:

The authors made no statement about the availability of the data underlying the findings described in the manuscript.

Response: Thank you for this important comment. We agree that the availability of the data underlying the findings was not clearly stated in the original submission. We have now added a Data Availability Statement in the revised manuscript in accordance with the PLOS ONE data policy. Because the study involves de-identified human participant data, the dataset cannot be shared publicly. We have clarified that requests for access to the minimal de-identified dataset should be directed to the appropriate institutional body of the original IRB-approving institution, subject to ethical and legal requirements.

[Revision: lines 302-307] Data Availability: The data underlying the findings of this study contain de-identified human participant data and cannot be shared publicly due to ethical and legal considerations related to participant confidentiality. Requests for access to the minimal de-identified dataset may be directed to the Institutional Review Board of Samsung Changwon Hospital, subject to institutional review and applicable regulations.

<Reviewer 2>

Materials and Methods

Comment 1: In line 74, where it states, "The study subjects include patients with RAS, BD, and PSS," it is necessary to specify how the diagnoses of these entities were carried out.

Response: Thank you for this important comment. We agree that the diagnostic definitions for each study group should be stated explicitly. We have revised the Materials and Methods section accordingly.

[Revision: lines 78-84] All diagnoses were established by board-certified rheumatologists. BD was defined according to the Japanese diagnostic criteria for Behcet’s disease [22], and PSS was classified according to the 2016 American College of Rheumatology (ACR)/European League Against Rheumatism (EULAR) classification criteria [23]. RAS was defined as recurrent oral ulceration without systemic features suggestive of BD or PSS and without fulfillment of corresponding diagnostic or classification criteria.

Comment 2: Describing the sample type, handling, and evaluation for the ANA and Anti-Ro tests is necessary given their significance in the results.

Response: Thank you for this important comment. We agree that the methodological details for ANA and anti-Ro testing should be described more explicitly because these variables were relevant to the interpretation of the results. We have revised the Materials and Methods section for the relevant sentences.

[Revision: lines 86-89] Antinuclear antibody (ANA) and anti-Ro antibody results were obtained from routine clinical laboratory testing in electronic medical records. Both tests were performed on serum samples, and positivity was determined according to the reference ranges and cut-off values used by the institutional laboratory at the time of testing.

Results

Comment 1: Since the results of interest in Table 1 focus on the Antinuclear Antibody Test (ANA) values, it is necessary to briefly explain how these results are interpreted to suggest the presence versus the possibility of an autoimmune disease.

Response: Thank you for this helpful comment. We agree that the ANA results shown in Table 1 require a brief interpretive context. We have revised the Results section to clarify that ANA was treated as a descriptive screening variable for autoimmune diseases, not as a classification item for PSS. We also noted that all PSS patients were positive for anti-Ro antibodies.

[Revision: lines 138-141] Although ANA is not included in the 2016 ACR/EULAR classification criteria for PSS, it is a basic screening test for autoimmune disease. In contrast, all PSS patients were positive for anti-Ro antibodies, consistent with the serologic item directly relevant to the classification criteria.

Comment 2: In paragraphs 150-156, the authors suggest that, based on their results, some metabolites are present in higher concentrations in patients with PSS compared to RAS and BD. They then present other metabolites that also show higher concentrations in PSS compared to RAS and BD. It would be necessary to explain why these are presented separately and not together, given that they refer to the same disease. Lines 154-156 suggest differences in metabolite levels but do not explain which specific metabolites are observed to be differentially expressed to distinguish between diseases, as presented in Figure 3.

Response: Thank you for your insightful comment. We agree that the original text was unnecessarily divided into two separate lists. We have merged these into a single description and revised the text to clarify the relationship between the metabolites identified in Figures 2 and 3.

[Revision: lines 166-173] As shown in Fig 2, it can be observed that the content of urea, fructose, succinic acid, malic acid, glucose, glycine, triethanolamine, L-serine, L-leucine, octadecanol, glutaric acid, L-proline, putrescine, nonanoic acid, dodecanoic acid, 3-hydroxypropanoic acid, cholesterol, aspartic acid, and myo-inositol is higher in PSS compared to RAS and BD. These findings indicate significant inter-disease differences in salivary metabolite levels. Notably, among these metabolites, aspartic acid and L-proline were also identified as key discriminatory metabolites in the PLS-DA analysis (Fig. 3), further supporting their potential as biomarkers for discriminating between RAS, BD, and PSS.

Comment 3: In paragraphs 158-161, it will be necessary to explain in more detail the results presented in Table 4, allowing for a clear understanding of the differences between diseases.

Response: Thank you for your comment. We would like to clarify that the detailed differences in metabolite levels among BD, PSS, and RAS based on Table 4 are already described in lines 178-183, where disease-specific elevations are specified for each group. We hope this adequately addresses your concern.

Comment 4: Develop the suggestion presented in lines 174-175, which indicates disease-dependent specific salivary metabolites.

Response: Thank you for this helpful suggestion. We have revised the corresponding sentence to more clearly describe disease-dependent salivary metabolites by specifying representative metabolites associated with BD, PSS, and RAS.

[Revision: lines 190-193] Collectively, these results indicate that specific salivary metabolites exhibit disease-dependent changes, with malonic acid and aspartic acid elevated in BD, L-proline increased in PSS, and cysteine elevated in RAS, suggesting their potential as non-invasive biomarkers for discriminating among RAS, BD, and PSS.

Comment 5: In lines 176-177, explain the differences in the regulation of metabolic pathways for each disease that support the discovery versus previous publications.

Response: Thank you for this insightful comment. While the Discussion already addressed metabolite-related biological functions, we have revised the corresponding section to more clearly emphasize differences in metabolic pathway regulation.

[Revision: lines 195-198] In particular, malonic acid and aspartic acid in BD are linked to oxidative stress and immune regulation [26-28], and putrescine and L-proline in PSS reflect anti-inflammatory responses and tissue repair [29-32].

Comment 6: Develop the presentation suggestion in lines 178-179.

Response: Thank you for this helpful suggestion. We have revised the corresponding sentence to provide a more explicit description of the potential clinical application of salivary metabolomics, particularly in the early differentiation of patients with nonspecific oral symptoms.

[Revision: lines 200-203] It may be particularly helpful in the early differentiation of patients presenting with nonspecific oral ulcers, where clinical symptoms overlap among BD, PSS, and RAS, potentially supporting timely diagnosis and reducing the need for invasive procedures such as biopsy.

1. Cui XX, Zhang LM, Su GN, Kijlstra A, Yang PZ. Specific sweat metabolite profile in ocular Behcet's disease. Int Immunopharmacol. 2021;97. doi: 10.1016/j.intimp.2021.107812. PubMed PMID: WOS:000679152100006.

2. Li Z, Mu Y, Guo CL, You X, Liu XY, Li Q, et al. Analysis of the saliva metabolic signature in patients with primary Sjogren's syndrome. Plos One. 2022;17(6). doi: 10.1371/journal.pone.0269275. PubMed PMID: WOS:000843567600028.

Attachment Submitted filename: Response_PLOS ONE_0403.docx

# Peer review: Decision Letter 1

**DOI:** 10.1371/journal.pone.0351403.r003

**Authors:** Ronell Bologna-Molina

6 May 2026

PONE-D-25-64353R1Metabolite profiling of saliva for the discrimination of Behcet’s disease, Sjögren’s syndrome, and recurrent aphthous stomatitisPLOS One

Dear Dr. Hwang,

Thank you for submitting your manuscript to PLOS ONE. After careful consideration, we feel that it has merit but does not fully meet PLOS ONE’s publication criteria as it currently stands. Therefore, we invite you to submit a revised version of the manuscript that addresses the points raised during the review process.

- **There are some minor points to elucidate, please perform the suggestion made by the reviewer.**

Please submit your revised manuscript by Jun 20 2026 11:59PM. If you will need more time than this to complete your revisions, please reply to this message or contact the journal office at plosone@plos.org. When you're ready to submit your revision, log on to https://www.editorialmanager.com/pone/ and select the 'Submissions Needing Revision' folder to locate your manuscript file.

Please include the following items when submitting your revised manuscript:

- A letter that responds to each point raised by the academic editor and reviewer(s). You should upload this letter as a separate file labeled 'Response to Reviewers'.
- A marked-up copy of your manuscript that highlights changes made to the original version. You should upload this as a separate file labeled 'Revised Manuscript with Track Changes'.
- An unmarked version of your revised paper without tracked changes. You should upload this as a separate file labeled 'Manuscript'.

If you would like to make changes to your financial disclosure, please include your updated statement in your cover letter. Guidelines for resubmitting your figure files are available below the reviewer comments at the end of this letter.

If applicable, we recommend that you deposit your laboratory protocols in protocols.io to enhance the reproducibility of your results. Protocols.io assigns your protocol its own identifier (DOI) so that it can be cited independently in the future. For instructions see: https://journals.plos.org/plosone/s/submission-guidelines#loc-laboratory-protocols. Additionally, PLOS ONE offers an option for publishing peer-reviewed Lab Protocol articles, which describe protocols hosted on protocols.io. Read more information on sharing protocols at https://plos.org/protocols?utm_medium=editorial-email&utm_source=authorletters&utm_campaign=protocols.

As the corresponding author, your ORCID iD is verified in the submission system and will appear in the published article. PLOS supports the use of ORCID, and we encourage all coauthors to register for an ORCID iD and use it as well. Please encourage your coauthors to verify their ORCID iD within the submission system before final acceptance, as unverified ORCID iDs will not appear in the published article. *Only* the individual author can complete the verification step; PLOS staff *cannot* verify ORCID iDs on behalf of authors.

We look forward to receiving your revised manuscript.

Kind regards,

Ronell Bologna-Molina

Academic Editor

PLOS One

**Journal Requirements:**

1. If the reviewer comments include a recommendation to cite specific previously published works, please review and evaluate these publications to determine whether they are relevant and should be cited. There is no requirement to cite these works unless the editor has indicated otherwise.

2. Please review your reference list to ensure that it is complete and correct. If you have cited papers that have been retracted, please include the rationale for doing so in the manuscript text, or remove these references and replace them with relevant current references. Any changes to the reference list should be mentioned in the rebuttal letter that accompanies your revised manuscript. If you need to cite a retracted article, indicate the article’s retracted status in the References list and also include a citation and full reference for the retraction notice.

[Note: HTML markup is below. Please do not edit.]

Reviewers' comments:

Reviewer's Responses to Questions

**Comments to the Author**

1. If the authors have adequately addressed your comments raised in a previous round of review and you feel that this manuscript is now acceptable for publication, you may indicate that here to bypass the “Comments to the Author” section, enter your conflict of interest statement in the “Confidential to Editor” section, and submit your "Accept" recommendation.

Reviewer #1: (No Response)

Reviewer #2: All comments have been addressed

**********

2. Is the manuscript technically sound, and do the data support the conclusions?

The manuscript must describe a technically sound piece of scientific research with data that supports the conclusions. Experiments must have been conducted rigorously, with appropriate controls, replication, and sample sizes. The conclusions must be drawn appropriately based on the data presented.

Reviewer #1: Partly

Reviewer #2: Yes

**********

3. Has the statistical analysis been performed appropriately and rigorously?

Reviewer #1: Yes

Reviewer #2: Yes

**********

4. Have the authors made all data underlying the findings in their manuscript fully available?

The PLOS Data policy requires authors to make all data underlying the findings described in their manuscript fully available without restriction, with rare exception (please refer to the Data Availability Statement in the manuscript PDF file). The data should be provided as part of the manuscript or its supporting information, or deposited to a public repository. For example, in addition to summary statistics, the data points behind means, medians and variance measures should be available. If there are restrictions on publicly sharing data—e.g. participant privacy or use of data from a third party—those must be specified.

Reviewer #1: Yes

Reviewer #2: Yes

**********

5. Is the manuscript presented in an intelligible fashion and written in standard English?

PLOS ONE does not copyedit accepted manuscripts, so the language in submitted articles must be clear, correct, and unambiguous. Any typographical or grammatical errors should be corrected at revision, so please note any specific errors here.

Reviewer #1: Yes

Reviewer #2: Yes

**********

6. Review Comments to the Author

Please use the space provided to explain your answers to the questions above. You may also include additional comments for the author, including concerns about dual publication, research ethics, or publication ethics. (Please upload your review as an attachment if it exceeds 20,000 characters)

**Reviewer #1:** Methods Section

Justifying sample size based on precedence from prior comparable studies can be scientifically acceptable, but it is generally considered a pragmatic or feasibility-based justification, and not the strongest form of statistical justification in rare diseases and exploratory metabolomics research.

This study revolves around 3 autoimmune diseases which are not common. There is however, not much statistical information on the prevalence of diseases to objectively prove their rarity, which could be a valid reason for justification of sample size based on prior comparable studies. Authors should please address this and provide what is known in the literature about the prevalence and rarity of these conditions objectively.

Authors should also explicitly indicate whether the study is an exploratory one or a confirmatory one. A clear indication that the study is an exploratory one or a hypothesis-generating one can provide a stronger scientific justification for the pragmatic determination of the sample size based on previous published metabolomics studies, because using previous studies alone is not equivalent to formal sample size calculation and does not provide adequate statistical power.

The study, being an exploratory one, has been clearly mentioned in the discussion, with the limitation on the small sample size clearly acknowledged. However, the type of study and the justification for the sample size should be clearly detailed in the methods section

**Reviewer #2:** All comments have been properly addressed and clarified in the work "Metabolite profiling of saliva for the discrimination of Behcet’s disease, Sjögren’s syndrome, and recurrent aphthous stomatitis"

**********

7. PLOS authors have the option to publish the peer review history of their article (what does this mean?). If published, this will include your full peer review and any attached files.

If you choose “no”, your identity will remain anonymous but your review may still be made public.

**Do you want your identity to be public for this peer review?** For information about this choice, including consent withdrawal, please see our Privacy Policy.

Reviewer #1: No

Reviewer #2: No

**********

[NOTE: If reviewer comments were submitted as an attachment file, they will be attached to this email and accessible via the submission site. Please log into your account, locate the manuscript record, and check for the action link "View Attachments". If this link does not appear, there are no attachment files.]

To ensure your figures meet our technical requirements, please review our figure guidelines: https://journals.plos.org/plosone/s/figures

You may also use PLOS’s free figure tool, NAAS, to help you prepare publication quality figures: https://journals.plos.org/plosone/s/figures#loc-tools-for-figure-preparation.

NAAS will assess whether your figures meet our technical requirements by comparing each figure against our figure specifications.

# Author comment: Author response to Decision Letter 2

**DOI:** 10.1371/journal.pone.0351403.r004

22 May 2026

Response to Reviewer 1: Thank you for this important comment. We agree that determining the sample size based on previous comparable studies represents a pragmatic and feasibility-based rationale rather than a formal statistical power calculation. In response, we have revised the Methods section to explicitly state the relevant contents with appropriate references.

Response to Reviewer 2: Thank you for the positive comment and for confirming that all previous concerns have been adequately addressed.

Attachment Submitted filename: Response to Reviewers_PONE-D-25-64353R1.docx

# Editor report: Decision Letter 2

**DOI:** 10.1371/journal.pone.0351403.r005

**Authors:** Ronell Bologna-Molina

28 May 2026

Metabolite profiling of saliva for the discrimination of Behcet’s disease, Sjögren’s syndrome, and recurrent aphthous stomatitis

PONE-D-25-64353R2

Dear Dr. Hwang,

We’re pleased to inform you that your manuscript has been judged scientifically suitable for publication and will be formally accepted for publication once it meets all outstanding technical requirements.

Within one week, you’ll receive an e-mail detailing the required amendments. When these have been addressed, you’ll receive a formal acceptance letter and your manuscript will be scheduled for publication.

An invoice will be generated when your article is formally accepted. Please note, if your institution has a publishing partnership with PLOS and your article meets the relevant criteria, all or part of your publication costs will be covered. Please make sure your user information is up-to-date by logging into Editorial Manager at Editorial Manager® and clicking the ‘Update My Information' link at the top of the page. For questions related to billing, please contact billing support.

If your institution or institutions have a press office, please notify them about your upcoming paper to help maximize its impact. If they’ll be preparing press materials, please inform our press team as soon as possible -- no later than 48 hours after receiving the formal acceptance. Your manuscript will remain under strict press embargo until 2 pm Eastern Time on the date of publication. For more information, please contact onepress@plos.org.

Kind regards,

Ronell Bologna-Molina

Academic Editor

PLOS One

Additional Editor Comments (optional):

Reviewers' comments:

# Editor report: Acceptance letter

**DOI:** 10.1371/journal.pone.0351403.r006

**Authors:** Ronell Bologna-Molina

PONE-D-25-64353R2

PLOS One

Dear Dr. Hwang,

I'm pleased to inform you that your manuscript has been deemed suitable for publication in PLOS One. Congratulations! Your manuscript is now being handed over to our production team.

At this stage, our production department will prepare your paper for publication. This includes ensuring the following:

* All references, tables, and figures are properly cited

* All relevant supporting information is included in the manuscript submission,

* There are no issues that prevent the paper from being properly typeset

You will receive further instructions from the production team, including instructions on how to review your proof when it is ready. Please keep in mind that we are working through a large volume of accepted articles, so please give us a few days to review your paper and let you know the next and final steps.

Lastly, if your institution or institutions have a press office, please let them know about your upcoming paper now to help maximize its impact. If they'll be preparing press materials, please inform our press team within the next 48 hours. Your manuscript will remain under strict press embargo until 2 pm Eastern Time on the date of publication. For more information, please contact onepress@plos.org.

You will receive an invoice from PLOS for your publication fee after your manuscript has reached the completed accept phase. If you receive an email requesting payment before acceptance or for any other service, this may be a phishing scheme. Learn how to identify phishing emails and protect your accounts at https://explore.plos.org/phishing.

If we can help with anything else, please email us at customercare@plos.org.

Thank you for submitting your work to PLOS ONE and supporting open access.

Kind regards,

PLOS ONE Editorial Office Staff

on behalf of

Professor Ronell Bologna-Molina

Academic Editor

PLOS One
